# The Effects of Methionine Acquisition and Synthesis on *Streptococcus Pneumoniae* Growth and Virulence

**DOI:** 10.1371/journal.pone.0049638

**Published:** 2013-01-22

**Authors:** Shilpa Basavanna, Suneeta Chimalapati, Abbas Maqbool, Bruna Rubbo, Jose Yuste, Robert J. Wilson, Arthur Hosie, Abiodun D. Ogunniyi, James C. Paton, Gavin Thomas, Jeremy S. Brown

**Affiliations:** 1 Department of Microbiology and Molecular Genetics, University of Texas-Houston Medical School, Houston, Texas, United States of America; 2 Department of Medicine, Centre for Inflammation and Tissue Repair, University College Medical School, Rayne Institute, London, United Kingdom; 3 Department of Biology (Area 10), University of York, York, United Kingdom; 4 Centro de Investigaciones Biologicas, CSIC and CIBER de Enfermedades Respiratorias (CIBERES), Madrid, Spain; 5 Division of Science, University of Bedfordshire, Park Square, Luton, Bedfordshire, United Kingdom; 6 Research Centre for Infectious Diseases, School of Molecular and Biomedical Science, The University of Adelaide, Adelaide, South Australia, Australia; University of Padova, Italy

## Abstract

Bacterial pathogens need to acquire nutrients from the host, but for many nutrients their importance during infection remain poorly understood. We have investigated the importance of methionine acquisition and synthesis for *Streptococcus pneumoniae* growth and virulence using strains with gene deletions affecting a putative methionine ABC transporter lipoprotein (Sp_0149, *metQ*) and/or methionine biosynthesis enzymes (Sp_0585 - Sp_0586, *metE* and *metF*). Immunoblot analysis confirmed MetQ was a lipoprotein and present in all *S. pneumoniae* strains investigated. However, vaccination with MetQ did not prevent fatal *S. pneumoniae* infection in mice despite stimulating a strong specific IgG response. Tryptophan fluorescence spectroscopy and isothermal titration calorimetry demonstrated that MetQ has both a high affinity and specificity for L-methionine with a K*_D_* of ∼25 nM, and a Δ*metQ* strain had reduced uptake of C_14_-methionine. Growth of the Δ*metQ*/Δ*metEF* strain was greatly impaired in chemically defined medium containing low concentrations of methionine and in blood but was partially restored by addition of high concentrations of exogenous methionine. Mixed infection models showed no attenuation of the Δ*metQ*, Δ*metEF* and Δ*metQ*/Δ*metEF* strains in their ability to colonise the mouse nasopharnyx. In a mouse model of systemic infection although significant infection was established in all mice, there were reduced spleen bacterial CFU after infection with the Δ*metQ*/Δ*metEF* strain compared to the wild-type strain. These data demonstrate that Sp_0149 encodes a high affinity methionine ABC transporter lipoprotein and that Sp_0585 – Sp_0586 are likely to be required for methionine synthesis. Although Sp_0149 and Sp_0585-Sp_0586 make a contribution towards full virulence, neither was essential for *S. pneumoniae* survival during infection.

## Introduction

The acquisition of essential nutrients from the host is a prerequisite for bacterial pathogens to be able to replicate and so to cause successful infection. Screens for virulence genes as well as targeted investigation of specific nutrient transporters has confirmed the importance of nutrient acquisition for the pathogenesis of infections for many microbial pathogens [1], [2], [3], [4], [5], [6], [7]. One group of nutrients required for bacterial growth are the amino acids, but there are only limited data on their importance for bacterial pathogenesis.

Methionine is one of the least abundant amino acids in physiological fluids (4 µg ml^−1^) [8], and yet is essential for protein synthesis and is a constituent of *S*-adenosylmethionine, the major biological methyl donor required for the biosynthesis of phospholipids and nucleic acids [9]. Two bacterial methionine transport systems have been described: the Methionine ABC (ATP binding cassette) Uptake Transporter (MUT) family [10], [11] and a secondary transporter termed BcaP [12]. In *Escherichia coli*, the MUT system is encoded by the *metD* locus and consists of the MetQ substrate binding protein (SBP), MetL transmembrane permease and the MetN cytoplasmic ATP-hydrolyzing protein (ATPase) [11]. *E. coli metD* mutants are unable to transport D-methionine or utilize this compound as a source of methionine [11]. Similar ABC transporters are the primary methionine transporters for *Bacillus subtilis (metNPQ*) [10], *Streptococcus agalactiae* (*metQ1NP*) [13] and *Streptococcus mutans* (*atmBDE*) [14]. The second methionine transport system, BcaP was described in *Lactococcus lactis* as a branched chain amino acids transporter, but is also involved in the transport of methionine [12]. Microorganisms and plants can also synthesize methionine by converting homoserine to homocysteine through addition of a sulphur group from either cysteine (requiring MetABC), sulfide (requiring MetA and CysD) or methionine using the SAM recycling pathway (MetK, Pfs, and LuxS) [15], [16]. Homocysteine is then methylated by methionine synthase (MetE) in conjunction with a methylenetetrahydrofolate reductase (MetF), with the methyl group supplied by 5-methyl tetrahydrofolate, to form methionine [15], [17]. Existing data show that methionine biosynthetic genes are required for the full virulence of *Brucella melitensis* [18], *Haemophilus parasuis* [19] and *Salmonella enterica* [20] and that mutation of the *S. agalactiae* methionine regulator MtaR attenuates virulence [8], suggesting methionine synthesis is essential for survival of many bacteria during invasive infection.

*Streptococcus pneumoniae* is a common nasopharyngeal commensal that is also an important pathogen frequently causing pneumonia, otitis media, septicaemia and meningitis. A recent investigation of the role of nine different ABC transporters for *S. pneumoniae* virulence identified an ABC transporter encoded by Sp_0148-52 that seemed to be important during pneumonia and septicaemia [7]. BLAST searches suggested this locus contained genes whose products have a high degree of identity to MetQNP and AtmBDE and therefore could be a *S. pneumoniae* methionine uptake ABC transporter. In this manuscript we describe in detail the Sp_0148-52 locus and the role of methionine during *S. pneumoniae* growth and virulence. Recombinant Sp_0149 was used to characterise the potential substrates of this ABC transporter, and deletion mutant strains of Sp_0149 and *metEF* were used to investigate role of methionine acquisition and synthesis during *S. pneumoniae* growth and virulence. In addition, as several *S. pneumoniae* lipoproteins have been shown to be effective vaccine candidates in animal models [21], [22], we also investigated the potential of recombinant Sp_0149 as a novel vaccine candidate.

## Results

### Results of BLAST alignments for Sp_0148-52

**Figure 1:**
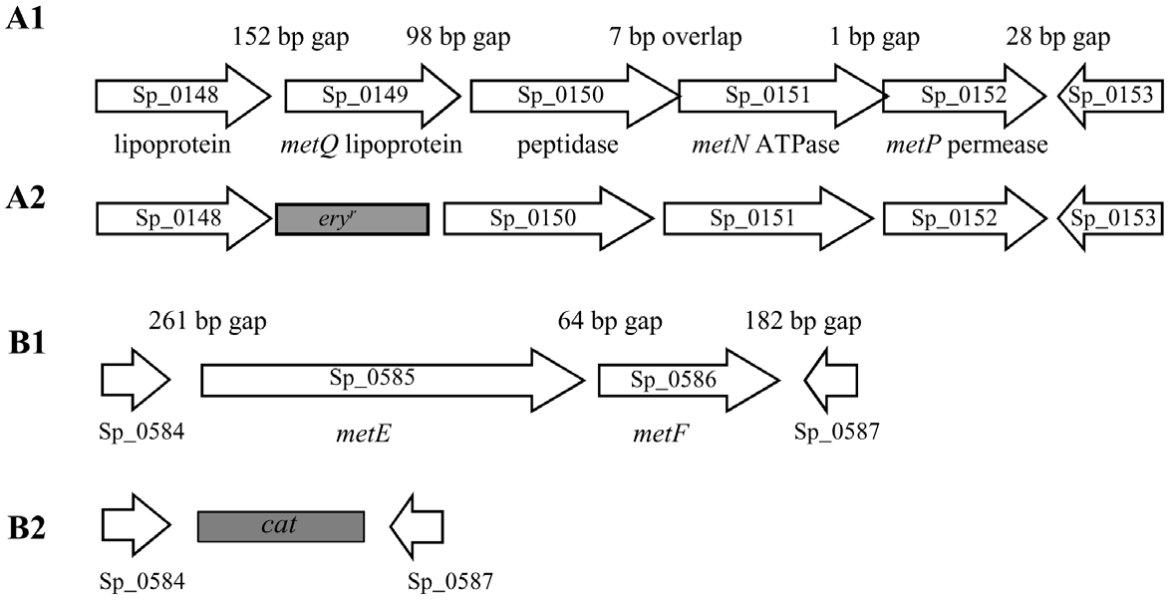
Schematic of the Sp_0148-53 and Sp_0584-0587 loci. (**A1**) Structure of the Sp_0148-53 locus. Each arrow represents one gene, with the Sp number stated within the arrow and gene annotation and probable function written below. The number of bp between the stop codon of the upstream gene and the ATG of the downstream gene is given above the arrows in the corresponding gap. (**A2**) Representation of the structure of the Sp_0148-53 locus in the Δ*metQ* strain; the erythromycin resistance cassette (*ery^r^*) is shaded grey. (**B1**) Structure of the Sp_0584-0587 locus, with the Sp number stated and gene annotation when available written below. The number of bp between the stop codon of the upstream gene and the ATG of the downstream gene is given above the arrows in the corresponding gap. (**B2**) Structure of the Sp_0584-0587 locus in the Δ*metEF* mutant strain, showing replacement of *metEF*genes with an in-frame copy of *cat* (shaded grey).

The Sp_0148-52 genetic locus was identified during a screen of ABC transporters for those involved in virulence. In this screen a mutant containing an insertion within Sp_0149 was attenuated in virulence in mouse models of pneumonia and sepsis [7]. The Sp_0148-52 region in the TIGR4 *S. pneumoniae* strain genome contains five genes and is 3669 bp in length (Fig. 1 A) [23]. Gene loci with identical organisation and high levels of identity at the amino acid level for the corresponding genes are present in *S. agalactiae* (SAN_1757-52) [13] and *S. mutans* (Smu.1942-38) [14] and contain the *metQNP* and *atmBDE* genes respectively that encode probable methionine ABC transporters. Both Sp_0148 and Sp_0149 possess potential lipoprotein signal sequences, LAACS (residues 21–25) and LAACG (residues 20–24) respectively, and are annotated in the TIGR4 genome as lipoproteins. Sp_0148 and Sp_0149 only share 21% identity and 34% similarity at the amino acid level, so are unlikely to have similar functions or have arisen by gene duplication. Sp_0148 has been annotated as an amino acid binding lipoprotein and is highly conserved amongst sequenced *S. pneumoniae* strains (98% identity at the amino acid level) and with proteins encoded by other streptococci (>49% identity). Sp_0149 is conserved in all the available *S. pneumoniae* genomes (>97% identity at the amino acid level) and has 62% identity at the amino acid level to the lipoprotein component of the *S. mutans* methionine transporter *atmBCDE* [14], suggesting it may have a similar function. BLAST searches demonstrate 73% identity at the amino acid level between the predicted proteins encoded by Sp_0151, *atmD* and *metN* (all predicted ATPases) and 70% for the predicted proteins encoded by Sp_0152, *atmE* and *metP* (all predicted permeases). Hence, Sp_0149-52 could encode a functional *S. pneumoniae* methionine ABC transporter, and therefore we have named Sp_0149 *metQ*, Sp_0151 *metN*, and Sp_0152 *metP* according to the nomenclature for the genes encoding a putative *S. agalactiae* methionine ABC transporter [13].

To investigate whether *S. pneumoniae metQNP* encode a methionine ABC transporter and the phenotype attributable to loss of its function, a *metQ* deletion mutant strain of 0100993 *S. pneumoniae* was constructed using a construct made by overlap extension PCR (Fig. 1A). Complete deletion of Sp_0149-0152 was not done as this would include deletion of Sp_0150 as well as *metQNP*. BLAST results suggested Sp_0150 encodes a protein belonging to the M20/M40/M45 family of peptidases. Genes encoding similar proteins are found within the *S. mutans* (*atmC*) and *S. agalactiae* (*pdsM*) methionine ABC transporter loci, but the function of the corresponding proteins is unknown. In addition the Sp_0148 lipoprotein may also utilise the MetN and MetP proteins to form an ABC transporter, making it difficult to attribute any phenotypes of an Sp_0149-0152 deletion mutant specifically to a single function. The correct identity of the mutant was confirmed using PCR and primers annealing to Sp_0148 or Sp_0150 and the erythromycin resistance gene *ery^r^* (data not shown). Attempts at complementing the *metQ* deletion mutant strain using reintegration of an intact copy of the full length gene were unsuccessful. However, qPCR demonstrated that expression of the downstream genes in the operon was not reduced by the Δ*metQ* mutation, with fold differences (SD) in transcript levels from the Δ*metQ* strain compared to the wild-type strain of 2.2 (2.3), 1.3 (1.2), and 1.1 (0.2) for Sp_0150, Sp_0151, and Sp_0152 respectively.

### Localisation studies of Sp_0149 lipoprotein

**Figure 2:**
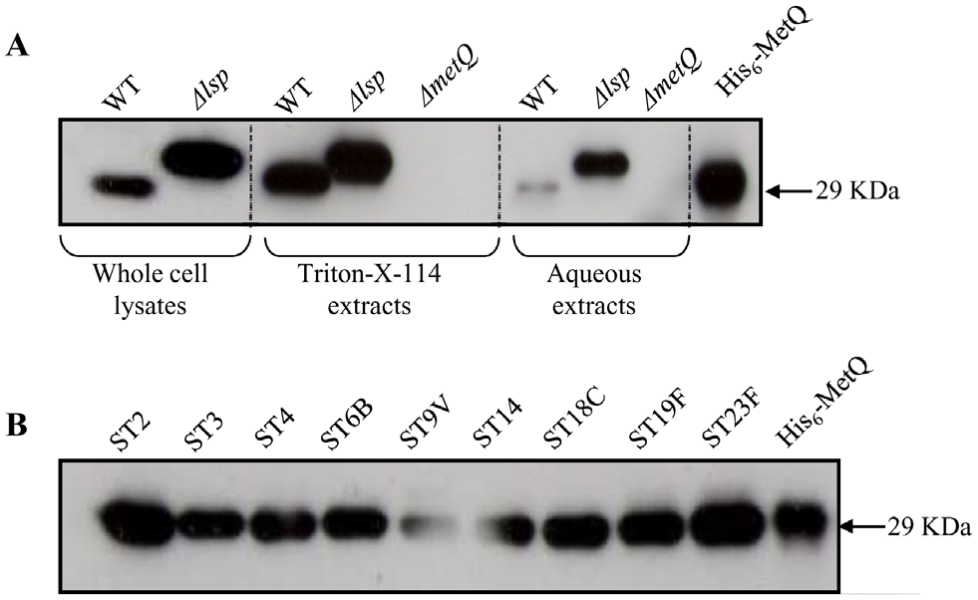
Immunoblots of *S. pneumoniae* using polyclonal anti-MetQ antibodies. (**A**) Probing of whole cell lysates, lipid and aqueous phases of Triton X-114 extracts of wild-type, Δ*lsp*, and Δ*metQ* 0100993 strains, and the recombinant MetQ protein (missing the N terminal signal sequence) with anti-MetQ antibodies. Equal numbers of bacteria were used for each strain, and the approximate sizes of the bands are displayed on the right. (**B**) Immunoblots of representative strains for common *S. pneumoniae* serotypes probed with anti-MetQ antibodies, showing the presence of a similar band in all strains.

To confirm whether the lipoprotein MetQ is localised to the *S. pneumoniae* cell membrane, polyclonal anti-MetQ antibodies were obtained by intraperitoneal inoculation of recombinant MetQ into mice. Serum obtained after 4 weeks from inoculated mice recognised recombinant MetQ protein (Fig. 2A and B), and immunoblots of whole cell wild-type *S. pneumoniae* lysates identified a single band of similar size to the predicted size of MetQ excluding the lipoprotein signal peptide sequence. The same band was present when Triton X-114 extracts of membrane bound proteins were probed with polyclonal anti-MetQ antibodies but was only present as a weak band in the aqueous fraction (Fig. 2A). Previous studies have demonstrated that cleavage of the N terminal lipoprotein signal peptide sequence of lipoproteins requires lipoprotein signal peptidase (Lsp), and that lipoproteins in the *S. pneumoniae* Δ*lsp* mutant are therefore slightly larger than in the wild type strain [24]. Immunoblotting of whole cell lysates and aqueous fractions of the Δ*lsp S. pneumoniae* strain with anti-MetQ serum demonstrated that, as predicted, the band representing MetQ was slightly larger than in the wild-type strain (Fig. 2A). The band representing MetQ in the aqueous extracts from the Δ*lsp* strain was also more prominent that in the wild-type strain, perhaps suggesting reduced retention of MetQ in the cell membrane in the absence of the Lsp enzyme. Overall, the immunoblot results demonstrate that MetQ is mainly associated with the membrane fraction and is processed by Lsp, confirming that MetQ is a lipoprotein.

### MetQ binds L-methionine with high affinity and is required for methionine uptake by *S. pneumoniae*

**Figure 3:**
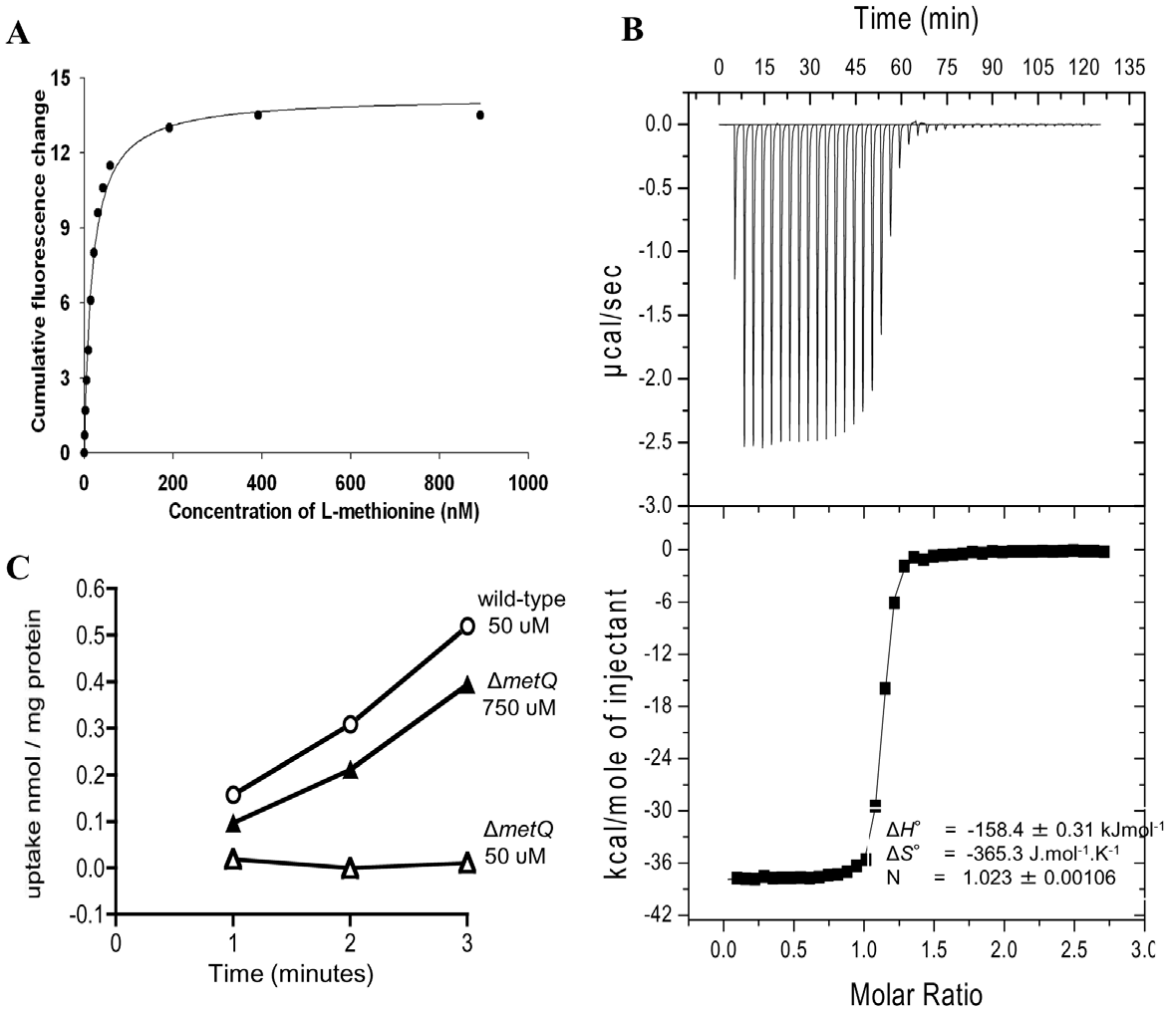
Analysis of L-methionine binding to MetQ. (A) Using tryptophan fluorescence spectroscopy: MetQ (0.05 µM) in 50 mM potassium phosphate pH 8 was excited at 280 nm at 37°C. Example titration of the MetQ signal change at 342 nm with increasing concentrations of L-methionine. (B) Binding isotherms for the interaction of MetQ with L-methionine. The top panel display heat changes upon injection of ligand and the low panel the integrated hears of injection (▪) and the best fit (solid line) to a single site binding model. (C) Radioactive uptake of ^14^C-methionine expressed as nmol/mg protein over time in minutes by the wild-type (circles, 50 µM of methionine added) and *ΔmetQ* (open triangles 50 µM of methionine added, filled triangles 750 µM of methionine added) *S. pneumoniae* strains.

Potential substrates for the MetQNP ABC transporter were investigated using fluorescence spectroscopy of recombinant MetQ (**Supplementary** **Figure 1**), a method widely used to investigate ligand specificity for SBPs due to the large conformational changes that occurs upon ligand binding [25], [26]. We observed an approximately 30% enhancement of the intrinsic fluorescence of the protein in the presence of L-methionine (Supplementary Fig. 2) and we determined a *K*_D_ of MetQ for L-methionine to be determined as 26.58±1.15 nM (Fig. 3A). We confirmed this tight binding using isothermal titration calorimetry, which yielded almost identical binding parameters of 26.55±1.28 nM (Fig. 3B), and also demonstrates 1∶1 binding of L-methionine to MetQ.

To investigate the specificity of MetQ for L-methionine, we examined ligand binding using a range of related ligands. Binding of D-methionine was around 1000 fold weaker with a *K*_D_ of 27.4±0.96 µM, while binding of DL-homocysteine was only around 250-fold weaker with a *K*_D_ of 7.0±0.26 µM (Supplementary Fig. 3). We also detected weak binding of α-methyl-DL-methionine with a *K*_D_ of 30.1±0.86 µM (Supplementary Fig. 3), but could not detect any binding for either L-cysteine of glycine (Supplementary Fig. 2). These data demonstrate that MetQ has a high affinity and specificity for L-methionine, and confirm that methionine is the probable major ligand for the *S. pneumoniae* MetQNP ABC transporter. To confirm that MetQ is required for methionine uptake by *S. pneumoniae*, uptake assays of ^14^C-labelled methionine were performed in chemically defined medium with the wild-type and Δ*metQ* strains [27]. As the mucoid phenotype of the serotype 3 strain prevents the efficient formation of bacterial pellets necessary for these assays, the Δ*metQ* mutation was transferred into a serotype 2 *S. pneumoniae* strain (D39). In C+Y medium without added methionine, the Δ*metQ* mutant strain was unable take up C_14_-methionine whereas the wild-type strain demonstrated significant uptake (Fig. 3C). In the presence of 750 µM methionine there was uptake of C_14_-methionine by the Δ*metQ* mutant strain suggesting the presence of a lower affinity methionine transport mechanism (**Fig. 3C**). These results confirm that *metQNP* is a methionine ABC transporter that is required for methionine uptake by *S. pneumoniae* when methionine availability is limited.

### Investigation of the lipoprotein MetQ as a vaccine candidate

**Figure 4:**
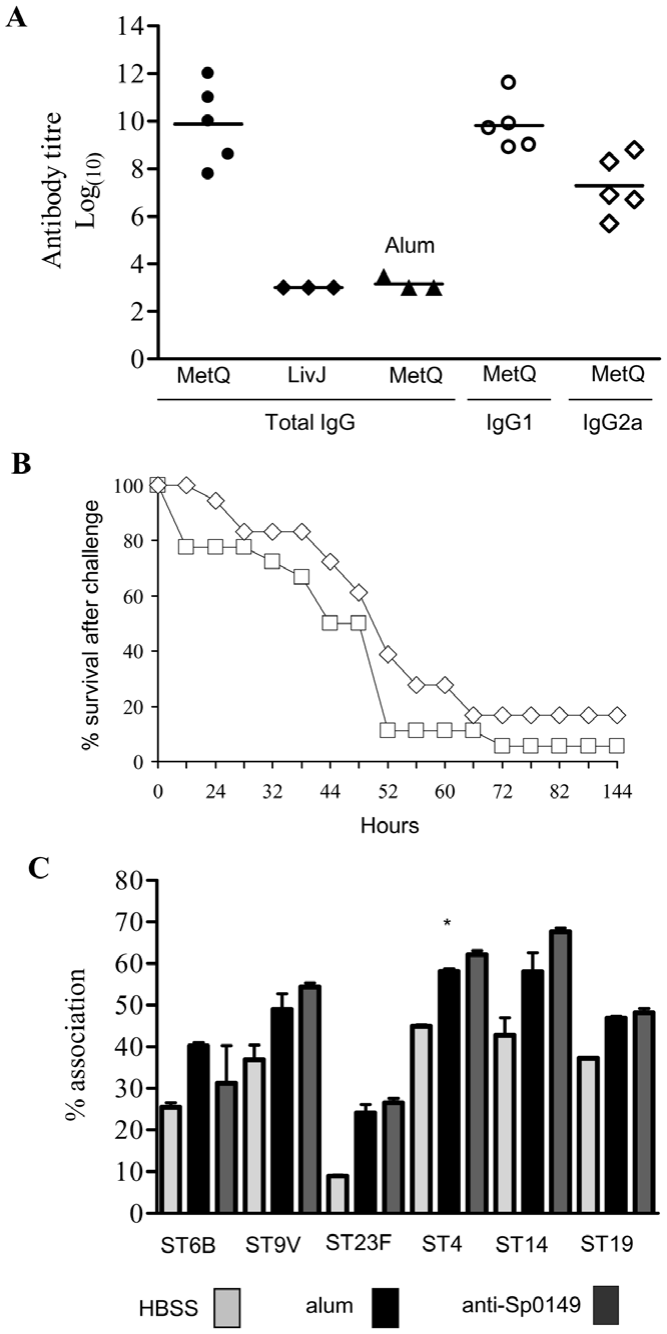
Investigation of MetQ as a vaccine candidate. (**A**) Mouse sera antibody titres to recombinant His-tagged MetQ after intraperitoneal immunisation with His_6_-MetQ. Target protein (His_6_-MetQ or the negative control SBP His_6_-LivJ) and IgG class are labelled along the X axis, and results were obtained using serum from His_6_-MetQ vaccinated mice with the exception of the filled triangles which represent results for serum from control mice (labelled alum). Filled symbols represent titres for individual mice for the total IgG titre, open circles for IgG1, and open diamonds for IgG2A titres. Bars represent median values. (**B**) Development of fatal disease in mice vaccinated with His_6_-MetQ (square symbols) and control (given alum and PBS alone, diamonds) mice after IP inoculation of 10^4^ CFU of D39 (n = 20). There were no statistical differences in survival (log rank test). (**C**) Phagocytosis of representative *S. pneumoniae* vaccine serotypes. Key: bacteria opsonised with: grey bars, HBSS; solid bars, serum from alum immunised mice; dark grey bars, polyclonal anti-MetQ sera from MetQ vaccinated mice.

**Figure 5:**
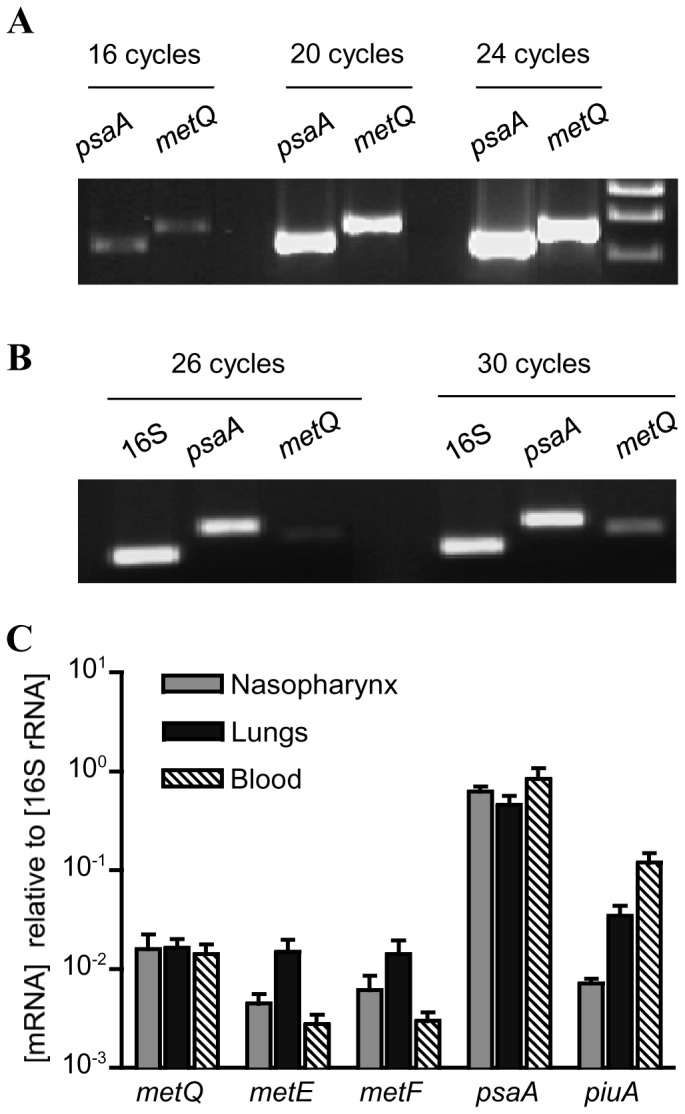
Gene expression of Sp_0149 during infection. (**A**) Ethidium stained agarose gel showing equal amplification efficiency for the primer pairs used for PCR of *psaA* and *metQ* when using *S. pneumoniae* 0100993 genomic DNA as the template. (**B**) Ethidium stained agarose gel of cDNA products generated by RT-PCR after 26 and 30 cycles using *S. pneumoniae* 0100993 total RNA extracted from the blood of infected mice and primers for 16S rRNA (internal control), *psaA* (positive control) and *metQ*. (C) Relative mRNA concentrations of selected genes of *S. pneumoniae* WCH43 (serotype 4) in various *in vivo* niches at 72 h post-intranasal infection of mice. Transcript abundance for each gene was obtained by microarray analysis, and normalized against those obtained for the 16S rRNA control. Quantitative fold differences for each transcript were determined as a ratio of its abundance to that of the 16S rRNA control. Data are means ± SEM for each gene transcript from three replicate challenge experiments.

Lipoprotein are attached to the external surface of bacterial membranes and have been shown to be effective vaccines that protect against *S. pneumoniae* infection in mouse models [21], [22]. Hence, MetQ was investigated as a potential novel vaccine candidate. PCR (data not shown) and immunoblots demonstrated that MetQ was present in **all of** a range of *S. pneumoniae* strains from common MLST sequence types for clinically important capsular serotypes (Fig. 2B). Mice were immunised by three intraperitoneal injections of 10 ug recombinant MetQ with alum as the adjuvant separated by 7 or 8 days, which resulted in significant serum titres of IgG (mainly IgG1) against MetQ that did not cross-react with the control lipoprotein LivJ (2) (Fig. 4A). However, when challenged by intraperitoneal inoculation with a potentially lethal dose of the serotype 2 *S. pneumoniae* strain D39 there were no differences in the speed of development of fatal infection and survival between vaccinated and control (given PBS and the adjuvant alone) mice (Fig. 4B). To investigate whether this lack of vaccine efficacy despite good anti-MetQ titres was due to lack of MetQ expression by *S. pneumoniae* during infection, semi-quantitative reverse transcriptase PCR (RT-PCR) for *metQ* transcripts was performed using *S. pneumoniae* RNA extracted from the blood of mice with severe *S. pneumoniae* septicaemia. As a positive control RT-PCR was also performed for *psaA*, which encodes another lipoprotein that is expressed during infection. RT-PCR readily identified a product of the right size for *metQ*, but at a lower level of expression compared to *psaA*, suggesting that relative lack of expression of *metQ* could be one reason why vaccination with the MetQ was not protective (Fig. 5B). These data were supported by transcriptome data calculating the relative abundance of *metQ*, *metE*, *metF*, *PsaA* and another lipoprotein *PiuA* at three anatomical sites during infection (Fig. 5C). The results show that transcript abundance for *metQ*, *metE* and *metF* were similar for bacteria found in the nasopharynx, lungs and blood. Importantly, the amount of *metQ* transcripts was almost two log_10_ lower and one log_10_ lower than the amount of *psaA* and *piuA* transcripts respectively, both of which encode effective lipoprotein vaccine candidates [28], [29], [30], [31]. These results support the hypothesis that lack of MetQ adundance during infection could explain the failure of vaccination with MetQ to provide protection against systemic disease. Furthermore, when assessed using an established flow cytometry assay polyclonal anti-MetQ sera did not significantly improve phagocytosis of other *S. pneumoniae* strains by a neutrophil cell line (Fig. 4C).

### In vitro phenotypes of the Δ*metQ*, Δ*metEF* and Δ*metEF*/Δ*metQ* strains

As synthesis of methionine may reduce the effects of loss of transport of methionine due to the *metQ* mutation on *S. pneumoniae* phenotype, additional mutant strains were constructed containing deletions of the *S. pneumoniae metE* and *metF* [32]. BLAST searches of *S. pneumoniae* TIGR4 genome identified the adjacent (and likely cotranscribed) genes Sp_0585 and Sp_0586 as homologues of the *S. mutans metE* and *metF* genes (47% and 38% identity at the amino acid level respectively) [14] (Fig. 1B). The genome structure suggested *metE* and *metF* were likely to be contranscribed independent of the adjacent genes, due to the large gap between Sp_0584 and Sp_0585, and because Sp_0587 is transcribed in the opposite direction to Sp_0586 (Fig. 1B). Deletion mutants replacing *metE and metF* with a chloramphenicol resistance marker were made in the wild-type (Δ*metEF*) or in combination with *metQ* deletion (Δ*metEF*/Δ*met*Q) *S. pneumoniae* 0100993 strains (Fig. 1B). The correct identity of the mutants was confirmed using PCR and primers annealing to Sp_0584 or Sp_0587 and the chloramphenicol resistance gene *cat* (data not shown).

**Figure 6:**
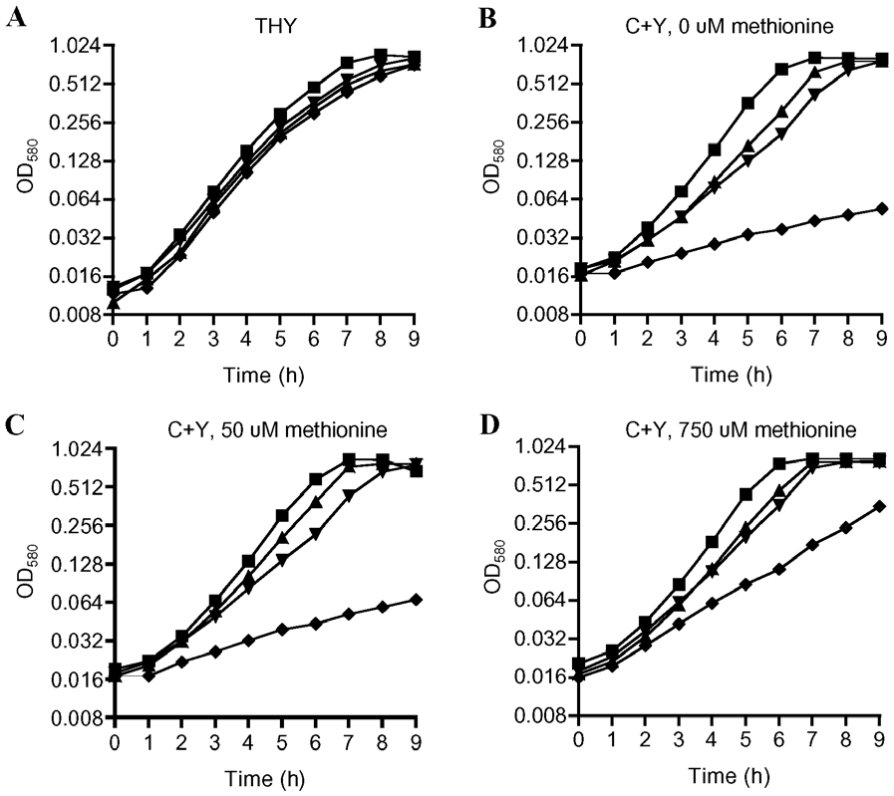
Growth of the wild-type, Δ*metQ*, Δ*metEF* and Δ*metEF*/Δ*metQ S. pneumoniae* strains in nutrient rich and semi defined media. Growth measured using OD_580_ of the wild-type (squares), Δ*metQ* (inverted triangles), Δ*metEF* (triangles) and Δ*metEF*/Δ*metQ* (diamonds) *S. pneumoniae* strains grown in THY (**A**), C+Y with no added methionine (**B**), C+Y with 50 µM methionine (**C**), or C+Y with 750 µM methionine (**D**). Each data point is the mean log_2_ OD_580_ for three samples.

To investigate the functional importance of methionine synthesis and uptake for *S. pneumoniae*, growth of the wild-type, Δ*metQ*, Δ*metEF* and Δ*metEF*/Δ*metQ* strains were compared in the complete medium THY and in the semi-defined medium C+Y with restricted methionine content. In THY there were no significant differences in growth between the wild-type and Δ*metQ*, Δ*metEF* and Δ*metEF/ΔmetQ* strains (Fig. 6A). In C+Y without added methionine (Fig. 6B), both Δ*metQ* and Δ*metEF* mutant strains had slightly impaired growth compared to the wild-type strain, but still reached similar growth levels to the wild-type strain after 9 hours. However, in C+Y with either no added methionine **or supplemented with only 50 µM of methionine** the double mutant Δ*metEF*/Δ*metQ* had markedly impaired growth **due to reduced generation times** (Fig. 6B and C). These results provide further evidence that MetQ is required for methionine uptake and indicate that MetEF are necessary for methionine synthesis. The impaired growth of the Δ*metEF*/Δ*met*Q strain in C+Y medium was partially restored by addition of 750 µM of methionine (Fig. 6D), suggesting there are additional **weaker** affinity secondary transport systems that allow the Δ*metEF*/Δ*met*Q strain to obtain methionine in the presence of high concentrations of substrate.

**Figure 7:**
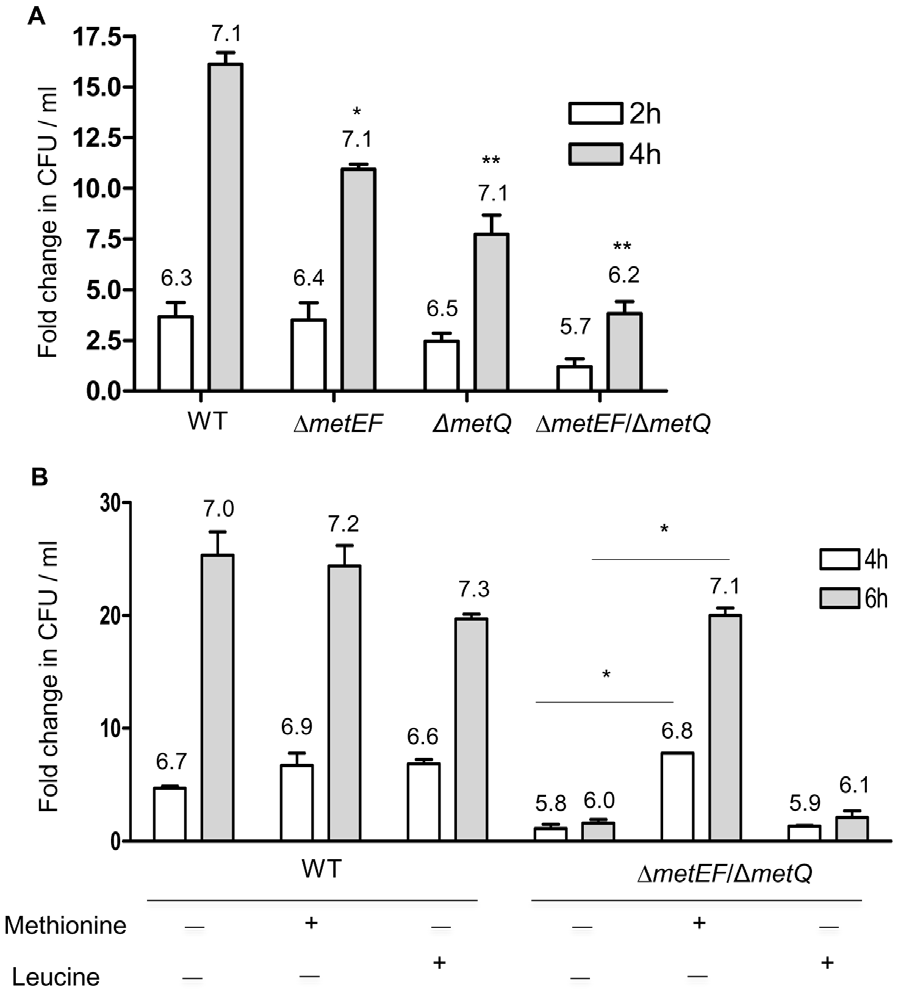
Growth of the wild-type, Δ*metQ*, Δ*metEF* and Δ*metEF*/Δ*metQ S. pneumoniae* strains in human blood or in human blood supplemented with amino acids. Data is presented as the mean (SD) fold change in the bacterial CFU per ml at 2 h (clear columns) and at 4 h (grey columns) in human blood (**A**) or at 4 h (clear columns) and 6 h (grey columns) in human blood supplemented with amino acids (**B**). *P* values for the differences between the wild-type and the mutant strains at 4 h (**A**) and between the Δ*metEF*/Δ*metQ* strain with and without added methionine at 4 h or 6 h (**B**) were obtained using ANOVA and post hoc tests, *P<0.05, ** *p*<0.01. **Log^10^ bacterial CFU per ml results for each condition are given in figures above the corresponding bar in the graphs.**

To assess the requirement for methionine for *S. pneumoniae* growth in physiologically relevant conditions, replication of these strains was assessed in freshly obtained human blood. The Δ*metQ* and Δ*metEF* strains were both significantly impaired in growth in blood (inoculum size 1.5×10^6^ CFU, 10.9 and 7.7 fold increase in CFU, respectively) compared to the wild type strain (inoculum size 0.5×10^6^ CFU, 16.2 fold increase, *P*<0.001, ANOVA) (Fig. 7A), **although all three strains reached similar total CFU per ml**. Moreover, the Δ*metEF*/Δ*met*Q strain had markedly reduced growth in blood with only 3.8 fold increase in CFU after 4 hours **and reaching a total CFU of nearly 1 log_10_ fewer than the wild-type strain** (Fig. 7A). Addition of 1 mM methionine but not leucine (a branched-chain amino acid not linked to the methionine biosynthetic pathways) largely reversed the growth defect of the double mutant in blood, without affecting growth of the wild-type strain (Fig. 7B). These results demonstrate that absence of methionine uptake and synthesis strongly impairs *S. pneumoniae* growth in physiological conditions found during infection.

### In vivo phenotypes of the Δ*metQ*, Δ*metEF* and Δ*metEF*/Δ*metQ* strains

**Figure 8:**
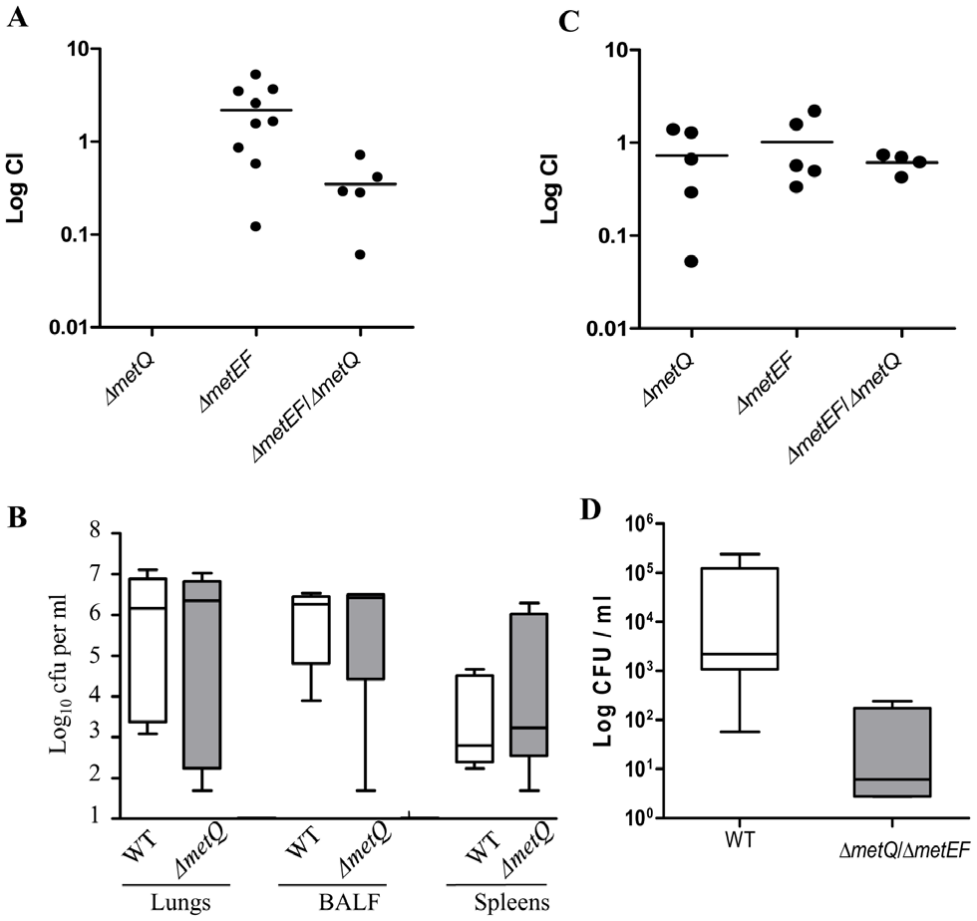
Virulence of the Δ*metQ*, Δ*metEF* and Δ*metEF*/Δ*metQ* strains in mouse models of infection. (**A**) CIs for mixed infections with the Δ*metQ*, Δ*metEF* and Δ*metEF*/Δ*metQ* and wild-type strains for bacteria recovered from the nasal washes 5 days after colonisation. Each point represents results for a single mouse, and bars median CIs for the group. (**B**) Log_10_ ml^−1^ bacterial CFU recovered from lungs, BALF and spleens of mice 24 hours after IN inoculation with **1×10^7^ CFU** of either the wild-type or Δ*metQ* strain. There were no statistical differences between the strains for any organ site. (**C**) Log_10_ ml^−1^ bacterial CFU recovered from blood 24 hours after IP inoculation with 3×10^3^ CFU of either the wild-type or Δ*metEF*/Δ*metQ* strain. *P* = 0.0317, Mann Whitney U test.

To investigate the effects of deletion of *metQ* and/or *metEF* on the ability of *S. pneumoniae* to colonise the nasopharynx, competitive indices (CIs) for mixed infection with the wild-type and the Δ*metQ*, or Δ*metEF* or Δ*metEF*/Δ*met*Q strains were determined in a mouse model of colonisation. None of the mutant strains were reduced in virulence in the colonisation model when CIs were calculated for nasal washes obtained 5 days after inoculation (Fig. 8A). Previously we have shown that a *metQ* disruption strain was attenuated in virulence in mouse models of sepsis and pneumonia when investigated using CIs [7]. To further assess whether the Δ*metQ* mutation affected the development of infection, groups of 5 CD1 mice each were intranasally inoculated with 10^7^ CFU of the wild-type or Δ*metQ* strain, and bacterial CFU from the BALF, lungs and spleens determined after 48 hours (Fig. 8B). There were no significant differences in the bacterial loads between the wild-type and Δ*metQ* strains in the BALF, lungs and spleens. Hence although previously we have shown disruption of *metQ* strain reduced virulence in a competitive model of lung infection [7], the Δ*metQ* strain was still able to cause significant infection when given as a pure inoculum. The severe growth defect of the double mutant Δ*metEF*/Δ*metQ* in human blood prompted us to investigate its growth in a mouse sepsis model. After intraperitoneal inoculation the number of Δ*metEF*/Δ*metQ* strain CFU recovered from the blood was approximately two log_10_ fewer than CFU recovered for the wild-type strain (*P* = 0.032, Mann Whitney U test) (Fig. 8C). These data suggested that the inability of the Δ*metEF*/Δ*metQ* strain to either transport or synthesise methionine has resulted in decreased but not completely abrogated virulence of this strain in a mouse sepsis model.

## Discussion

In this study, we provide evidence that the *S. pneumoniae* Sp_0148-52 locus encodes a methionine uptake ABC transporter and that Sp_0585 and Sp_0586 encode MetE and MetF required for methionine biosynthesis. BLAST searches demonstrated that the Sp_0148-52 and Sp_0585/Sp_0586 encode proteins with high degrees of similarity to *S. mutans* AtmBDE and MetEF respectively, which have been shown to be required for methionine uptake and synthesis [13], [14]. Deletion of the gene encoding the lipoprotein component Sp_0149 (which we have called *metQ*) resulted in a strain that had reduced growth in methionine restricted media and no detectable uptake of radioactive methionine. Tryptophan fluorescence spectroscopy confirmed MetQ bound to L-methionine with high affinity as well as D-methionine and homocysteine, an amino acid that is related to methionine. Deletion of Sp_0585/Sp_0586 increased the growth defect of the *metQ* deletion strain in methionine restricted media and in blood, supporting a role for the products of these genes for methionine synthesis. Genetic complementation would be helpful to link the *metEF* and *metQ* mutations to these phenotypes but was not possible to achieve, as is often the case with *S. pneumoniae*. However the successful biochemical complementation of the in vitro phenotypes by exogenous methionine and the lack of impaired transcription of the rest of the operon in the *metQ* mutant strongly support that the observed phenotypes were caused by the mutations. Overall the data indicate that *S. pneumoniae* methionine acquisition and synthesis is largely dependent on the Sp_0148-52 locus and Sp_0585 and Sp_0586 loci respectively. Unlike the equivalent *S. mutans* mutants [14], the *S. pneumoniae metQ* and *metEF* mutant strains were still able to grow in the absence of supplementation with methionine. This observation and the persisting growth of the *S. pneumoniae ΔmetQ/metEF* double mutant in media containing high concentrations of methionine suggest that there are additional mechanisms for *S. pneumoniae* methionine uptake, possibly similar to BcaP of *L. lactis* [12]. One candidate is the Sp_0481-4 locus which contains an upstream consensus sequence for the streptococcal methionine regulator MtaR/MetR (data not shown).

As well as encoding the MetQNP methionine ABC transporter, the Sp_0148-52 locus also encodes another SBP (Sp_0148) unrelated to MetQ and a peptidase (Sp_0150). The functions of these proteins and why their genes are associated with the *metQNP* genes are unknown. The peptidase could be required for the breakdown of extracellular peptides to provide substrates for MetQNP, but this hypothesis will require further investigation. Previously it has been suggested that two separate zinc uptake lipoproteins AdcA and AscAII share the same permease and ATPase components [33]. Similarly, the Sp_0148 SBP could associate with MetN and MetP to make an ABC transporter with separate affinities to the MetQ lipoprotein, perhaps another amino acid as Sp_0148 has some similarities to proteins annotated as glutamine SBPs.

Existing vaccines against *S. pneumoniae* are unable to provide protection against all strains, prompting the investigation of surface exposed antigens such as SBPs that are generally highly conserved between different *S. pneumoniae* strains as novel vaccine candidates. Existing data show that several ABC transporters SBPs are effective vaccines in animal models of *S. pneumoniae* [28], [30], [34], [35]. However, vaccination with MetQ did not prevent fatal systemic infection despite inducing a reasonable antibody response and the surface location of MetQ. RT-PCR and **transcriptome data** suggested that expression of *metQ* during actual infection was **considerably** lower than that of the SBP vaccine candidates *psaA* and *piuA* [36]; this low level of expression could readily contribute to the lack of protective efficacy of high titres to the *S. pneumoniae* MetQ. In addition, unlike IgG raised against other SBPs [31], [34], [35], anti-MetQ antibodies did not promote neutrophil phagocytosis of *S. pneumoniae*, suggesting these antibodies are functionally ineffective at promoting immunity.

Several *S. pneumoniae* ABC transporters have been shown to be required for full virulence, including those involved in the acquisition of metals (manganese, zinc, and iron), phosphate, polyamines, and branched chain amino acids [5], [6], [7], [29], [34], [35], [37]. As methionine is essential for bacterial growth and has restricted availability in mammalian systems, with a concentration of 4 µg ml^−1^ in physiological fluid, the MetQNP ABC transporter might also be expected to be important for *S. pneumoniae* virulence. Indeed, our previous data using mixed infection experiments with the wild-type strain and a *metQ* mutant created by insertional mutagenesis showed the *metQ* mutant strain was significantly less competitive in both pneumonia and sepsis infection models [7]. **However, the competitive nature of the mixed infection model makes it a very stringent test of virulence for mutant strains, and** in this manuscript we have shown that when given a pure inoculum of the deletion Δ*metQ* or wild-type strains the progression of infection in mouse model of pneumonia was very similar. These results are similar to those we have described for the *S. pneumoniae* branched chain amino acid ABC transporter, which also has reduced virulence when competing against the wild-type strain but could still establish fatal infection [7]. The double mutant Δ*metEF/metQ* strain was attenuated in virulence in a sepsis model with lower bacterial CFU than the wild-type strain, suggesting that methionine synthesis helped to maintain virulence of the Δ*metQ* strain. **However, somewhat surprisingly the mutant Δ** ***metEF/metQ*** **strain was still able to establish systemic infection in mice, despite its poor growth in blood in vitro and a circulating methionine level in mammalian blood of 7 to 43 µM, a level that is associated with a strong growth defect for the this strain in medium**. Similarly, there was no evidence that deletion of either *metQ* or *metEF* individually or in combination affected colonisation when assessed using highly sensitive competitive infection assays. **These results may indicate that local methionine availability at the site of infection is relatively high, perhaps due to cell lysis,** and adequate for *S. pneumoniae* growth even in a strain containing deletions affecting both high affinity methionine uptake and methionine biosynthesis. **The effect on virulence of some** ***S. pneumoniae*** **mutations vary with strain background, and it is possible that in other strains** ***metQ*** **and** ***metEF*** **deletion will impair virulence to a greater extent than seen in the serotype 3 strain used for these studies**.

To conclude, we have assessed the role of the MetQNP ABC transporter and MetEF methionine synthesis enzymes for *S. pneumoniae* growth and virulence. The results demonstrate that MetQNP is required for methionine uptake, and that dual mutation of *metQ* and the *metEF* locus has a profound effect on growth of *S. pneumoniae* in methionine restricted conditions. Methionine uptake and synthesis assisted, but was not essential, for the development of *S. pneumoniae* infections. These data provide more information on the micronutrient requirements of *S. pneumoniae* during the development of infection.

## Materials and Methods

### Ethics statement

All animal experiments were approved by the UCL Biological Services Ethical Committee and the UK Home Office (Project Licence PPL70/6510). Experiments were performed according to UK national guidelines for animal use and care, under UK Home Office licence.

### Bacterial strains, media and growth conditions

The *E. coli* strain M15 (Qiagen) were used for the cloning of Sp_0149 lipoprotein gene. Capsular serotypes 2 D39 strain and capsular serotype 3 *S. pneumoniae* 0100993 strain were used to construct *S. pneumoniae* mutant strains for the *in vitro* and *in vivo* phenotype analysis [1]. A range of *S. pneumoniae* strains from common multi-locus sequence typing (MLST) sequence types (STs) for clinically important capsular serotypes were kind gifts from Professor Spratt, Imperial College [38]. *E. coli* was cultured at 37°C using Luria Bertani (LB) broth or agar plates and *S. pneumoniae* strains were cultured in the presence of 5% CO_2_ at 37°C on Columbia agar (Oxoid) supplemented with 5% horse blood (TCS Biosciences), or in Todd-Hewitt broth supplemented with 0.5% yeast-extract (Oxoid) or C+Y media [39]. Plasmids and mutant strains were selected by using appropriate antibiotics (carbenicillin 100 µg ml^−1^ and kanamycin 25 µg ml^−1^ for *E. coli*, 0.2 µg ml^−1^ erythromycin for *S. pneumoniae*). Stocks of *S. pneumoniae* were stored as single use 0.5 ml aliquots of THY broth culture (O.D_580_ 0.3–0.4) at −70°C in 10% glycerol. Growth of *S. pneumoniae* strains in broth was monitored by measuring optical density at 580 nm (OD_580_). **Replication of** ***S. pneumoniae*** **strains in freshly obtained human blood was determined by inoculating with 0.5 to 2.0×10^6^ CFU ml^−1^ (depending on strain and experiment). After 2, 4, 6 h of growth at 37°C under 5% CO_2_, serial dilutions were plated on to blood agar plates to enumerate the CFU. Fold change was calculated by dividing the results by the inoculum CFU**.

### Nucleic acid manipulations, reverse transcriptase PCR and transcriptome analysis

*S. pneumoniae* genomic DNA was extracted from bacteria grown in THY using a modified Wizard genomic DNA kit (Promega) and RNA using SV total RNA extraction kit (Promega) as previously described [6]. Total RNA from *S. pneumoniae* found in mouse blood was extracted as described by Ogunniyi *et al* [40]. *S. pneumoniae* were harvested from human and mouse blood by brief centrifugation at 825 *g* at 4°C for 5 minutes. The resulting supernatant was centrifuged further at 15500 *g* at 4°C for 5 minutes, and the bacterial pellet resuspended in 300 µl of pre-warmed (65°C) acid-phenol (Ambion) and incubated for 5 minutes at 65°C followed by further addition of 300 µl of pre-warmed NAES buffer and incubation at 65°C for 5 minutes with intermittent mixing. The mixture was cooled on ice for 1 minute and centrifuged at 15500 *g* for 1 minute, and the aqueous phase was re-extracted twice with acid-phenol and NAES buffer followed by twice with 300 µl of chloroform. To this mixture, sodium acetate was added at a final concentration of 300 mM followed by addition of 2 volumes of ethanol and RNA was precipitated at −20°C overnight, centrifuged at 6000 rpm for 5 minutes and washed in 70% ethanol before resuspension in 50 µl of nuclease-free water. To the resulting RNA, recombinant RNasin ribonuclease inhibitor (Promega N251A) was added to a final concentration of 1 U µl^−1^ and then treated with 0·5 U µl^−1^ RQ1 RNase-free DNase (Promega M610A) at 37°C for 40 minutes. Aliquots of this RNA were stored at −70°C until use. An aliquot was used to check the purity by RT-PCR with and without reverse transcriptase using gene specific primers. **qPCR was performed as previously described** [**24**] **using cDNA amplified from 0.4 µg of total RNA using the Applied Biosystems Geneamp RNA PCR core kit (Applied Biosystems, UK), and target-specific primers used for Sp_0150, Sp_0151 and Sp_0152 expression (** **Table 1** **). For each gene, crossing point (Cp) values were determined from the linear region of the amplification plot and normalized by subtraction of the Cp value for 16S RNA generating a ΔCp value. Relative change was determined by subtraction of the ΔCp value for the wild-type strain from the ΔCp value for the mutant strain (ΔΔCp value), and fold change calculated using the formula 2^−ΔΔCp^. Transcriptome data for RNA extracted from** ***S. pneumoniae*** **serotype 4 strain WCH43 recovered from mouse nasopharynx, lungs and blood was obtained as described** [**41**] **, using whole-genome** ***S. pneumoniae*** **PCR arrays obtained from the Bacterial Microarray Group at St George's Hospital Medical School, London, England (** http://bugs.sghms.ac.uk/ **). Transcript abundance for each gene was obtained, and quantitative fold differences determined as a ratio of a specific gene abundance to that of the 16S rRNA control**.

**Table 1:** Strains, plasmids and primers used in this study. Restriction enzyme sites in primers 5′ linkers are underlined.

Name	Description (source/reference)
Plasmids	
pID701	Shuttle vector for IDM transformation of *S. pneumoniae*: Cm^r^ (1)
pPC111	pID701 containing an internal portion of Sp_0149 in the *Xba*I site: Cm^r^ (this study)
ΔmetQ	pGEM-Teasy containing a deletion of Sp_0149: Amp^r^ (this study)
pPC138	pQE30UA carrying full length Sp_0149: Km^r^, Amp^r^ (this study)
Strains	
0100993	*S. pneumoniae* capsular serotype 3 clinical isolate (2)
D39	*S. pneumoniae* capsular serotype 2
TIGR4	*S. pneumoniae* capsular serotype 4
JSB6B	*S. pneumoniae* capsular serotype 6B (42)
JSB9V	*S. pneumoniae* capsular serotype 9V (42)
JSB14	*S. pneumoniae* capsular serotype 14 (42)
JSB18C	*S. pneumoniae* capsular serotype 18C
JSB19F	*S. pneumoniae* capsular serotype 19F
JSB23F	*S. pneumoniae* capsular serotype 23F (42)
Δ*metQ*	0100993 containing the Sp_0149 deletion construct: Ery^r^ (this study)
Δ*metQ^D39^*	D39 containing the Sp_0149 deletion made with Δ*metQ* genomic DNA: Ery^r^ (this study)
Δ*metEF*	0100993 containing the Sp_0585and Sp_0586 deletion construct: Cm^r^ (this study)
Δ*metQ/*Δ*metEF*	0100993 containing the *metQ* and *metEF* deletion constructs: Ery^r^ and Cm^r^ (this study)
Expression and qPCR Primers	
16s.1	GGT GAG TAA CGC GTA GGT AA
16s.2	ACG ATC CGA AAA CCT TCT TC
PsaA.1	CGT TCC GAT TGG GCA AGA C
PsaA.2	GCA CTT GGA ACA CCA TAG
Sp0149.1	GGC TCT TGC AGC TTG CGG
Sp0149.2	GGC TTT CGT TTG TAG CGT C
Sp0150.F	GAGCTATACAGCGCCCTTTG
Sp0150.R	CGCTGGCACAGTGTCATAGT
Sp0151.F	ACCGTGTTGCAGTTATGCAG
Sp0151.R	CCATGGCTTCGTCAATACCT
Sp0152.F	GGAGCTGGTGGTATCGGTAA
Sp.0152.R	TCTCCCAAGAATTGGATTGC
Mutation primers	
Sp0148F	CAC CAA TTG CCC AAA ATC C
Ery-Sp0148R	TATT TTAT ATT TTT GTT CAT GAT TCT TTC TCC TTA AAA ATA
Ery-Sp0150	ATT ATT TAA CGG GAG GAA ATA A TAA GAA ACA GGG AGG TGG GAG
Sp0150R	CCA AGG CAT TTT TGG TCC C
EryF	ATGAACAAAAATATAAAATA
EryR	TTATTTCCTCCCGTTAAATAAT
Sp0583F	GCGTGGGACAGTCCGACAATG
Cm-Sp0584R	CATTATCCATTAAAAATCAAAGATGTGTCCTCCAAAATTTGTTGTTG
Cm-Sp0587F	CTAATGACTGGCTTTTATAATAAAAGCAAACCATTCTTCTCAGG
Sp0587R	CTCACGACCCGCAAAAGTC
Cm.1	TTATAAAAGCCAGTCATTAG
Cm.2	TTTGATTTTTAATGGATAATG

Restriction digests, ligation of DNA fragments, fractionation of DNA fragments by electrophoresis, and transformation of *E. coli* (by heat shock) were performed according to established protocols [42]. DNA fragments were purified from electrophoresis gels using the QIAquick gel extraction kit. RT-PCR was performed using the Access RT-PCR system (Promega) and gene specific primers (Table 1). The NCBI website (http://www.ncbi.nlm.nih.gov/blast) was used to perform BLAST searches and alignments of the available complete and incomplete bacterial nucleotide and protein databases. Primers for both PCR and RT-PCR were designed using sequences displayed by the ARTEMIS software.

### Construction of Δ*metQ*, Δ*metEF* and Δ*metEF*/Δ*met*Q *S. pneumoniae* mutant strains

Strains, plasmids and primers used for this study are described in Table 1. Target genes were amplified by PCR from *S. pneumoniae* 0100993 genomic DNA using primers designed from the TIGR4 genome sequence (http://www.tigr.org) [23]. The Δ*metQ* strain was constructed by overlap extension PCR [43] using a transformation fragment in which the Sp_0149/*metQ* gene had been replaced by the erythromycin resistance cassette *ery*. Two products corresponding to 630 bp 5′ (primers Sp0148F and Ery-Sp0148R) and 672 bp 3′ (primers Ery-Sp0150F and Sp0150R) to *metQ* were amplified from *S. pneumoniae* genomic DNA by PCR carrying 3′ and 5′ linkers complementary to the 5′ and 3′ portion of the *ery* gene respectively. *ery* was amplified from pACH74 using PCR and the primers EryF and EryR [24]. Similarly, For the in-frame deletion of *metEF* (Sp_0585-Sp_0586), a construct was created in which 921 bp of flanking DNA 5′ to the Sp_0585 ATG (primers Sp0583F and Cm-Sp0584R) and 725 bp of flanking DNA 3′ to the Sp_0586 ORF (primers Cm-Sp0587F and Sp0587R) were amplified by PCR from *S. pneumoniae* genomic DNA and fused with the chloramphenicol resistance marker (*cat*, amplified from pID701, a suicide vector containing *cat* gene, with primers CmF and CmR) by overlap extension PCR. The constructs were transformed into *S. pneumoniae* by homologous recombination and allelic replacement using competence stimulating peptide (CSP-1) (kind gift from D. Morrison) and selection with antibiotics according to established protocols [1], [5]. Deletion of *metQ* and/or *metEF* was confirmed by PCR and sequencing of the PCR products (performed by Lark Technologies Inc. UK or UCL sequencing services using the Big Dye™ Terminator technique and gene specific PCR primers).

### Cloning, expression and purification of His_6_-MetQ

Recombinant MetQ protein was expressed in *E. coli* and purified using an N terminal His-tagged QIAexpressionist™ system. Primers Sp0149.1 and Sp0149.2 amplified a full-length *metQ* from the *S. pneumoniae* D39 strain (excluding the 5′ portion encoding the predicted N-terminal signal peptide), which was then ligated into the pQE30 expression vector to make the plasmid pPC138 and transformed into *E. coli* strain M15. **For synthesis of recombinant proteins, the transformed cells were grown aerobically in 5 ml of Lennox broth (LB) for 8 h, and used to inoculate 50 ml LB for overnight aerobic growth at 37°C. This was then used to inoculate 625 ml of LB at 30°C. Cells were allowed to grow aerobically to an** ***A*** **_650_ of 0.4–0.6 before production of recombinant protein was induced with 1 mM isopropyl 1-thio-β-D-galactopyranoside (IPTG), followed by four hours of incubation at 30°C. Cells were harvested by centrifugation at 4430× g for 15 min at 4°C and resuspended in buffer A (50 mM potassium phosphate, pH 7.8, 500 mM NaCl, 10 mM immidazole and 20% glycerol). The cells were lysed by sonication and cell debris was removed by centrifugation at 38000× g for 30 min at 4°C and the supernatant containing overexpressed protein was collected. Nickel affinity chromatography was used for the purification of the hexahistidine tagged protein using 5 ml His-trap column [GE Healthcare]. The soluble fraction containing the over produced protein was passed over column pre-equilibrated with buffer A. Weakly bound contaminants were removed by washing the column with 20 CV of buffer A containing 40 mM imidazole and the recombinant protein was eluted with elution buffer containing 500 mM imidazole. Purification products were analysed by SDS-PAGE gel and shown to contain 99% pure protein of the expected size for His_6_-MetQ. Purified protein was dialysed in 50 mM potassium phosphate pH 7.8 and concentrated using Vivaspin 5 kDa MWCO concentrators. Protein concentration was determined from the absorbance at 280 nm (** ***A*** **_280_) using a calculated molar extinction coefficients of 45780 cm^−1^ M^−1^ for MetQ.** His_6_-Sp0749 (LivJ) was also purified using an N terminal His-tagged QIAexpressionist™ system as described in [7].

### Preparation of ligand free His_6_-MetQ

**Binding proteins often co-purify with their cognate ligands and thus must be made ligand free in order to check binding with alternate ligands in vitro. Co-purified ligand was removed from MetQ using guanidinium HCl (GdmHCl) as described previously** [**44**] **. The protein was partially unfolded while bound to the His-trap column, by including additional wash steps (in buffer A with 30 mm imidazole) of 40 CV with 2 m GdmHCl, 4 CV with 1.5 m GdmHCl, 4 CV with 1 m GdmHCl, 4 CV with 0.5 m GdmHCl, and finally 8 CV with 0 m GdmHCl. The protein was eluted with elution buffer as described previously.**

### Immunoblots and Triton-X-114 extract preparation

*S. pneumoniae* were grown in THY medium until the OD_580_ reached 0.6 and the cells were centrifuged, the pellet resuspended in sterile phosphate buffered saline (PBS) and sonicated. The cell lysate was centrifuged, and the supernatants used for further analysis by immunoblotting. Lipid associated proteins were extracted according to Khandavilli *et al* [24] using Triton X-114. Protein samples from Triton X-114 extracts were separated on SDS-PAGE 10% and 12% resolving gels respectively, blotted onto nitrocellulose membranes and probed with polyclonal mouse antisera raised against MetQ (1∶2500 dilution) according to standard protocols [45].

### Enzyme Linked Immunosorbent assay (ELISA)

ELISA was performed according to Jomaa *et al* [31]. Microtitre ELISA plates were coated with 100 µl of antigen at a concentration of 5 µg ml^−1^ in TSA buffer and incubated overnight at 4°C. The plates were washed 5 times with ELISA wash buffer and soaked in 150 µl of 2% BSA-Tween for 2–4 hours at 37°C and washed again for 3–4 times with ELISA wash buffer. A 1/1000 dilution of mouse serum was made in BSA-Tween diluent buffer and added to the first well, and then 2 fold dilutions transferred to subsequent wells and incubated at 37°C for 4 hours. The wells were then washed 6 times with ELISA wash buffer and incubated overnight at 4°C in 100 µl of 1/15000 dilution of goat anti-mouse IgG conjugated to alkaline phosphate (Sigma) diluted in enzyme diluent. The ELISA plate was then washed 5 times in ELISA wash buffer and 100 µl of dinitrophenol (dNP) substrate solution added to each well and incubated for 1 hour at 37°C (dNP substrate solution was prepared by adding 1 tablet of dNP (Sigma) in 5 ml of water). The absorbance was read at 405 nm and antibody titre was calculated as the lowest dilution giving an OD equal to or greater than 0.3.

### Opsonophagocytosis assays

To assess the effect of anti-metQ antibodies on the interaction of *S. pneumoniae* with phagocytes, we measured the proportion of a neutrophil cell line associated with fluorescent bacteria using a previously described flow cytometry opsonophagocytosis assay [31], [46]. The complement source used was commercially available baby rabbit serum (Sigma S7764; rabbit HLA-ABC). *S. pneumoniae* strains were fluorescently labelled by incubation with 5, 6-carboxyfluorescein-succinimidyl ester (FAM-SE; Molecular Probes, Eugene, Oreg.) solution (10 mg/ml in dimethyl sulfoxide; Sigma) in 0.1 M sodium bicarbonate buffer for 1 h at 37°C and then washed six times with Hanks balanced salt solution (HBSS) in 0.2% bovine serum albumin and stored in aliquots at −70°C in 10% glycerol (approximately 10^9^ CFU/ml). The human cell line HL-60 (promyelocytic leukemia cells; CCL240; American Type Culture Collection, Manassas, Va.) was used to provide the effector cells after differentiation into granulocytes by using previously described protocols. Differentiated HL60 cells were harvested by centrifugation (160× *g*, 8 min, 4°C) and washed twice with HBSS and once with HBSS in the presence of Ca^2+^ and Mg^2+^. FAM-SE-labelled bacteria (10^6^ CFU) were opsonized with 1/100, 1/40, and 1/10 dilutions of serum in a 96-well plate for 20 min at 37°C with horizontal shaking (170 rpm). Negative controls were included, using the same volume of HBSS. HL60 cells (10^5^) were added to the opsonized bacteria in the microplate plate and incubated for 30 min at 37°C with shaking, after which the bacteria and cells were fixed using 3% paraformaldehyde and analyzed using flow cytometry. A minimum of 6,000 cells per sample were analyzed.

### ^14^C-methionine uptake assays

Radioactive uptake assays were performed by the rapid filtration method as previously described [27] with minor modifications. *S. pneumoniae* strains were grown in C+Y medium until the OD_620_ reached 0.2–0.4. These experiments were performed using a capsular serotype 2 *S. pneumoniae* strain (D39) as the mucoid colonies of the capsular serotype 3 strain prevented effective pelleting of the bacteria for these assays. Bacteria were harvested at 13000 *g* for 20 minutes and resuspended in 50 mM potassium phosphate buffer (pH 7.2) with 1 mM MgCl_2_ to an OD_620_ between 0.8–1.1. Uptake of methionine was determined in 1 ml assays containing 0.85 ml of bacterial cells and a final concentration of 50 **or 750 µM** methionine with 0.125 µCi ^14^C-methionine (GE Healthcare, United Kingdom). Samples (150 µl) containing bacteria, radioactive and non-radioactive substrates were removed at time intervals (0, 1, 2, and 3 minutes) and immediately filtered through glass fibre filters (Whatman GF/F), washed twice with 50 mM potassium phosphate buffer, and then washed filters placed in scintillation vials in 5 ml of Ready Safe scintillation cocktail (Beckman Coulter) and radioactivity determined using a Wallac 1214 RackBeta liquid scintillation counter. Bichinchoninic acid protein assay (Sigma Aldrich, UK) determined that an optical density (620 nm) of 1 was equivalent to 0.238 mg protein, and this figure was used to convert the radioactivity counts to nmol solute per mg protein.

### Fluorescence Spectroscopy

**Protein fluorescence experiment used a FluoroMax 4 fluorescence spectrometer (Horiba Jobin-Yvon) with connecting water bath at 37°C. Ligand free protein (MetQ) was used at a concentration of 0.05 µM in 50 mM potassium phosphate, pH 7.8. Protein was excited at 281 nm with slit widths of 5 nm and emission was monitored at 352 nm with slit widths of 5 nm. To examine alternative ligands for MetQ, potential ligands were added at concentrations up to 5 mM. To determine the** ***K_D_*** **for ligand binding, the protein was titrated with increasing concentrations of the ligand and the corresponding fluorescence change was monitored in time acquisition mode. The cumulative fluorescence change was plotted in SigmaPlot and the** ***K_D_*** **was calculated from the hyperbolic fit of the binding curve. L-methionine, D-methionine, DL-Homocysteine, α-Methyl-DL-methionine, L-Cysteine and Glycine were purchased from Sigma-Aldrich.**

### Isothermal Titration Calorimetry

**Calorimetry experiment was performed using a VP-ITC instrument (MicroCal Inc., GE Heath Sciences) by taking 1.4 ml of ligand free MetQ in the calorimeter cell and 400 µl of L-methionine ligand in the syringe. The concentration of ligand in the syringe was typically 10 times that in the cell, whereas the cell concentration was chosen according to** ***c*** **value of 50, where** ***c*** **= [macromolecule]/(predicted)** ***K_D_*** **. Experiments were carried out in 50 mM potassium phosphate buffer, pH 7.8, at 37°C. Both cell and syringe solutions were degassed at 35°C for 10 min before use. The titrations were performed as follows. A single preliminary injection of 3 µl of ligand solution was followed by 40 injections (6 µl), delivered at an injection speed of 10 µl s^−1^. Injections were made at 3-min intervals with a stirring speed of 307 rpm. Raw titration data were integrated and fit to a one-site model of binding using MicroCal Origin version 7.0.**

### Animal models of infection models

Infection experiments were performed in age and sex matched groups of outbred CD1 mice (Charles River Breeders) between 4 to 8 weeks old. For mixed infections, equivalent numbers of bacteria from stocks of wild-type and mutant *S. pneumoniae* strains were mixed and diluted to the appropriate concentration. For the nasopharyneal colonisation model, 10^7^ CFU of bacteria in 10 µl were administered by intranasal inoculation under halothane general anaesthesia, and nasal washes were obtained 5 days post colonisation. Serial dilutions of the samples were plated onto Columbia blood agar plates containing optochin (50 µg ml^−1^) and/or gentamycin (5 µg ml^−1^) to differentiate pneumococcus from other contaminating streptococci and to enumerate CFU. The CI was calculated using the formula: ratio of mutant to wild-type strain recovered from mice divided by the ratio of mutant to wild-type strain in the inoculum [47]. A CI of less than 1 indicates the mutant strain is attenuated in virulence compared to the wild-type strain, and the lower the CI the more attenuated the mutant strain. Experiments were performed using pure inocula of wild-type or mutant strains to calculate bacterial CFU in recovered target organs for each strain at specific time points [47]; for the systemic model of infection 1×10^3^ CFU bacteria in 100 µl were inoculated by intraperitoneal (IP) injection and spleen homogenates obtained at 24 hours for plating [5], [6], [24]; and for the pneumonia model, 5×10^6^ CFU bacteria in 40 µl were given by intranasal (IN) inoculation under halothane general anaesthesia, and lung homogenates obtained at 48 hours for plating [6], [24]. For the vaccination infections, mice were given by IP injection 100 µg of MetQ or just PBS mixed with 10% alum on three occasions separated by 7 to 8 days. Serum was obtained for immunoblots and antibody titres ELISAs from five mice four weeks after the last vaccination, and the remaining vaccinated mice challenged by IP inoculation with 1×10^4^ *S. pneumoniae* D39 strain CFU. Disease progression was monitored using previously established criteria to identify mice likely to progress to fatal disease [5].

### Statistical analysis

All the *in vitro* growth curves were performed in triplicates and represented as means and standard deviations. Results of growth curves, radioactive uptake and binding assays were analysed using two-tailed *t*-tests. Target organ CFU were compared between strains using the Mann Whitney U test, and the survival of infected mice using the log rank test.
